# An Immune Microenvironment-Integrated Intestinal-on-a-Chip Model for Investigating Immunopathogenesis in Inflammatory Bowel Disease

**DOI:** 10.3390/molecules31091520

**Published:** 2026-05-03

**Authors:** Shiyang Ying, Huanhua Xu, Yi Xu, Xianqiang Mi

**Affiliations:** 1 School of Microelectronics, Shanghai University, Shanghai 200444, China; yingshiyang@shu.edu.cn; 2 State Key Laboratory for the Modernization of Classical and Famous Prescriptions of Chinese Medicine, Jiangxi University of Chinese Medicine, Nanchang 330004, China; huanhua323@jxutcm.edu.cn; 3 Shanghai Institute of Microsystem and Information Technology, Chinese Academy of Sciences, Shanghai 200050, China; 4 School of Physics and Optoelectronic Engineering, Hangzhou Institute for Advanced Study, University of Chinese Academy of Sciences, Hangzhou 310024, China; 5 University of Chinese Academy of Sciences, Beijing 100049, China; 6 Human Phenome Institute, Fudan University, Shanghai 200438, China

**Keywords:** inflammatory bowel disease, intestinal-on-a-chip, drug evaluation

## Abstract

Owing to the multifactorial nature of inflammatory bowel disease (IBD) pathogenesis, conventional two-dimensional (2D) models inadequately recapitulate the complex in vivo microenvironment. This study sought to develop an immune-microenvironment-integrated intestinal-on-a-chip model to overcome these limitations. A microfluidic chip was engineered to co-culture intestinal epithelial (Caco-2) cells and macrophages, facilitating the simulation of IBD pathological conditions for mechanistic investigations. Following inflammatory stimulation, M0 macrophages polarized into the M1 phenotype, concomitant with the upregulation of pro-inflammatory cytokines, including tumor necrosis factor-alpha (TNF-α), interleukin-6 (IL-6), and interleukin-1 beta (IL-1β). This induction disrupted the expression of tight junction proteins (e.g., zonula occludens-1 [ZO-1]) in Caco-2 cells, thereby compromising epithelial barrier integrity. Infliximab was used as a model drug to inhibit TNF-α and modulate macrophage polarization within the chip, effectively rescuing impaired epithelial barrier integrity. This study establishes a reliable intestinal-on-a-chip model that recapitulates macrophage–epithelial interactions in IBD, providing a robust platform for elucidating the mechanisms underlying intestinal barrier dysfunction and developing targeted therapeutic strategies.

## 1. Introduction

IBD is a chronic inflammatory disorder defined by persistent inflammation of the colonic mucosa and dysregulated immune responses [1]. Clinically, IBD manifests as rectal bleeding, abdominal pain, and progressive weight loss, with severe cases advancing to life-threatening complications, including toxic megacolon (a life-threatening dilation of the colon) and fulminant colitis [2,3]. Epidemiological data from China reveal an increasing prevalence ranging from 11.4 to 22 per 100,000 population, underscoring the escalating burden of IBD [4,5]. Current therapeutic strategies are predominantly palliative, centering on symptom management to sustain partial remission, as gaps in understanding IBD’s complex pathophysiology impede the development of curative therapies [6]. Tumor necrosis factor (TNF) inhibitors, the first-line biologic therapy for moderate-to-severe IBD, exhibit suboptimal efficacy: Approximately 30–40% of patients are primary non-responders, and an additional 23–46% experience secondary loss of response within one year of treatment [1,7]. This therapeutic gap has established IBD prevention and treatment as a high-priority area for innovation in the food, consumer goods, and pharmaceutical sectors [8]. The challenge in developing targeted therapies stems from the incomplete elucidation of the pathological mechanisms underlying intestinal inflammation [9]. Current evidence supports a multifactorial etiology, wherein exogenous triggers (e.g., intestinal microbiota dysbiosis, infectious agents) interplay with host factors (e.g., intestinal epithelial barrier dysfunction, genetic susceptibility) to initiate and perpetuate chronic inflammation [10,11]. In response, the scientific community is pursuing the development of comprehensive intestinal microbiome models and personalized modeling strategies to dissect these interactions [12]. However, in vitro modeling requires a delicate balance: cellular models must be simplified, stable, and reproducible while preserving physiological relevance to accurately recapitulate the core pathological features of IBD [13]. Immune dysregulation represents a central pathological axis in IBD, characterized by the infiltration of innate (neutrophils) and adaptive (lymphocytes) immune cells into the intestinal lamina propria, aberrant autoantibody production, and cytokine network imbalances [14]. Notably, dysregulated polarization of M0 macrophages plays a pivotal role in driving inflammatory cascades, although the precise mechanisms underlying this dysregulation remain incompletely elucidated [15]. The complexity of the IBD immune microenvironment—including crosstalk between immune cells, stromal cells, and the intestinal microbiota—poses significant challenges to elucidating specific pathogenic pathways, necessitating further integrative research [16,17].

Traditional research models, such as static 2D cell line cultures, inadequately recapitulate the three-dimensional (3D) architecture of the intestinal mucosa, its dynamic fluid milieu, and complex intercellular crosstalk [18]. Animal models, while valuable for certain applications, are constrained by species-specific physiological discrepancies, prohibitive costs, and ethical limitations that hinder translational relevance [19]. For instance, 2D cell cultures exhibit inherent instability in maintaining anaerobic conditions or long-term co-culture systems, thereby compromising their capacity to authentically replicate the immunopathological microenvironment of IBD [20,21]. These methodological constraints significantly impede mechanistic investigations into IBD pathogenesis and the development of targeted therapies [22,23]. Ex vivo-cultured human intestinal tissue explants, particularly those obtained from IBD patients, offer superior fidelity in replicating in vivo structural and functional characteristics, enabling critical insights into patient-specific heterogeneities (e.g., immune dysregulation patterns and differential drug responsiveness) [24]. Nevertheless, such models remain hampered by two principal limitations: first, sample scarcity; and second, limited survival time in vitro (even under optimal conditions, survival is limited to a maximum of 24 h) [25,26].

Microfluidic organ-on-a-chip (OoC) platforms have emerged as a promising alternative to conventional models due to their capacity for precise physiological simulation at the organ level [27]. These systems employ three key technical components—microfluidic perfusion networks, multicellular co-culture architectures, and dynamic mechanical stimulation (including shear stress and cyclic tensile forces)—to reconstruct the complex 3D microenvironment of human intestinal tissues with unprecedented fidelity [28,29]. Notably, the integrated biomechanical cues enable authentic replication of intestinal epithelial–mesenchymal interactions and immune cell trafficking dynamics, thereby establishing a robust in vitro model for studying mucosal immunology and inflammatory responses [30].

Although significant progress has been made with existing intestinal-on-a-chip models, most models still focus on the epithelial barrier or microbial interactions, and the simulation of immune cell-specific interactions (such as those involving macrophages) remains inadequate [31]. Studies have shown that the proportion of M1 macrophages in the colonic mucosa of IBD patients is significantly elevated, while the proportion of M2 macrophages is reduced; simultaneously, related inflammatory factors (such as IL-6, TNF-α, and IL-1β) are abnormally active, suggesting that macrophages play a key role in the pathogenesis of IBD [32]. Therefore, the development of the intestinal-on-a-chip capable of integrating the immune microenvironment is crucial for elucidating the pathogenesis of IBD [32,33,34].

Based on this, this study designed and developed an intestinal-on-a-chip model by introducing Caco-2 cells to establish an intestinal model. Additionally, it established an immunologically active microenvironment containing M0 macrophages and simulated the inflammatory response of IBD. By measuring the secretion levels of pro-inflammatory cytokines in the chip, the inflammatory status can be well reflected. Finally, we demonstrated the potential of our system for drug testing by treating an inflamed model with Infliximab—one of the most widely used TNF blockers for treating IBD.

## 2. Results

### 2.1. Establishment of the Intestinal-on-a-Chip Model

**Figure 1:**
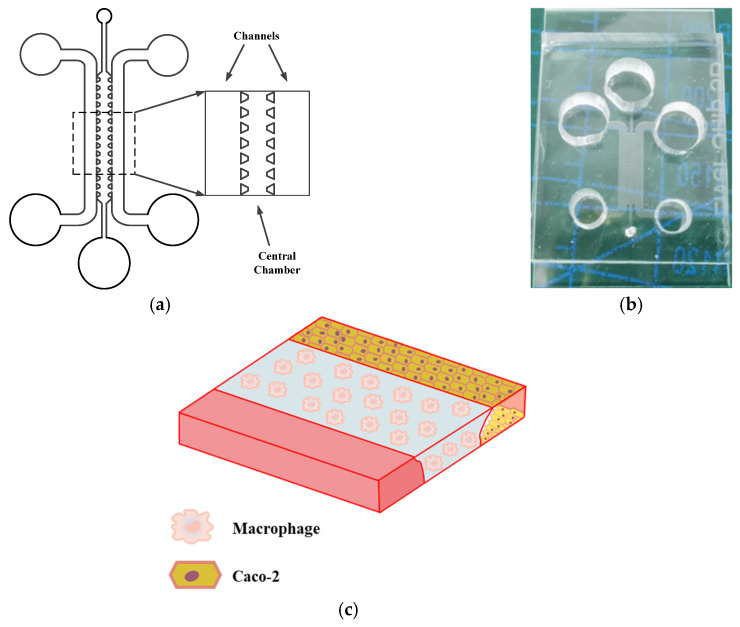

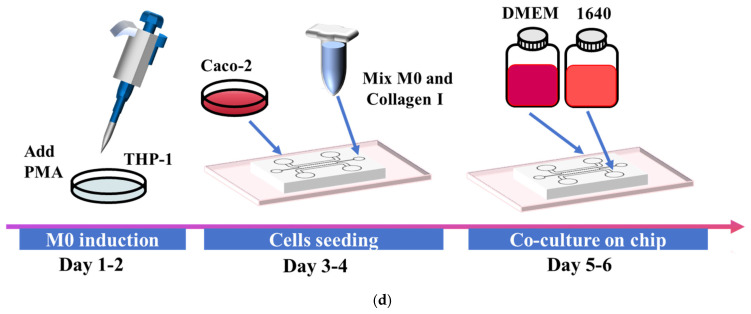
The schematic diagram and physical layout of the chip used for creating the intestinal barrier model. (**a**) Chip layout overview. (**b**) Photograph of the fabricated chip. (**c**) Schematic diagram of cell seeding on the chip. (**d**) Experimental timeline for the formation of the model.

As shown in Figure 1a,b, an intestinal-on-a-chip was fabricated from optically transparent, flexible polydimethylsiloxane (PDMS) polymer and consists of three parallel hollow microchannels. The central channel and the two lateral channels are separated by uniformly arranged micropillars. As shown in Figure 1c, macrophages, key immune cells implicated in the pathogenesis of IBD, were loaded into the central channel. Caco-2 cells were seeded in one lateral channel, while the culture medium was perfused through the other lateral channel to provide nutrients.

### 2.2. Development of the Intestinal Barrier Model

#### 2.2.1. Sequential Establishment of the Intestinal Barrier Model

The cell seeding process of the intestinal-on-a-chip is shown in Figure 1d. Construction of all models commenced with the uniform injection of a collagen type I (Collagen I) gel into all three microchannels. First, THP-1 monocytes were differentiated into M0 macrophages using phorbol 12-myristate 13-acetate (PMA). M0 macrophages were then uniformly suspended at a defined density within a Collagen I solution, which was injected into the central channel to form an immune cell-embedded matrix layer. The liquid matrix filled the channels via capillary action; the array of trapezoidal microcolumns prevented the gel from leaking into adjacent channels during the subsequent gelation process. Following complete gelation of the central matrix, a low-concentration Collagen I solution was introduced into the two side channels. After the second gelation, place 5 μL of Caco-2 cell suspension with a cell density of 1 × 10^6^ cells/mL at the inlet of the lateral channel. The chip was then oriented vertically, allowing gravity to draw the cells from the inlet into the channel, thereby facilitating Caco-2 cell adhesion to the gel surface. Meanwhile, 300 μL of culture medium was added to the inlets of the two lateral channels but not added to the outlets, allowing gravity to drive the medium to flow gradually from the inlet to the outlet and thus creating continuous perfusion within the channel to provide a continuous nutrient supply to the immune cell and the Caco-2 cell. Then, the Caco-2 cells proliferated and formed a confluent monolayer covering the channel surface, thereby generating a tight intestinal barrier. Ultimately, an intestinal-on-a-chip model exhibiting stable phenotypic characteristics and high reproducibility was successfully constructed.

#### 2.2.2. Characterization of the Intestinal-on-a-Chip

**Figure 2:**
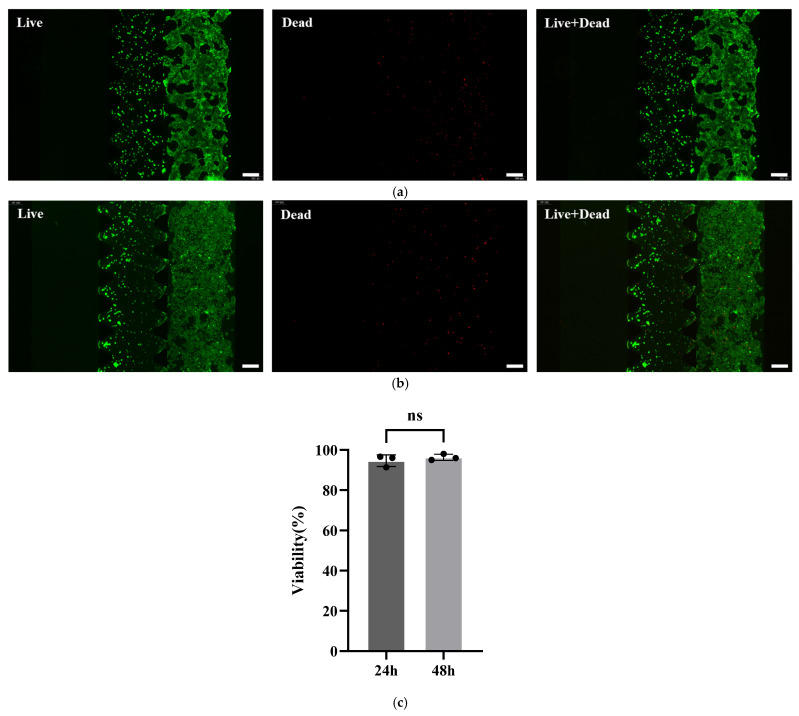
Representative images of live and dead cell staining at the cultivation time of (**a**) 24 h and (**b**) 48 h. Live cells with calcein-AM and dead cells with propidium iodide. Scale bars: 200 μm. (**c**) Viability of Caco-2 cells after 24 h and 48 h of cultivation. Data was acquired from the intestinal epithelial cell channel of three individual models (n = 3). Values are means ± SD. Statistical analysis by *t*-tests (ns: not significant).

We evaluated the biocompatibility of the intestinal-on-a-chip via cell viability assays, as shown in Figure 2a,b. Live/dead staining was performed on Caco-2 cells cultured for 24 h and 48 h within the chip. As shown in Figure 2c, quantitative image analysis revealed that the average viability of Caco-2 cells on the chip reached 94.65% after 24 h of culture and 96.34% after 48 h, confirming the excellent compatibility of the chip system for Caco-2 cell growth.

**Figure 3:**
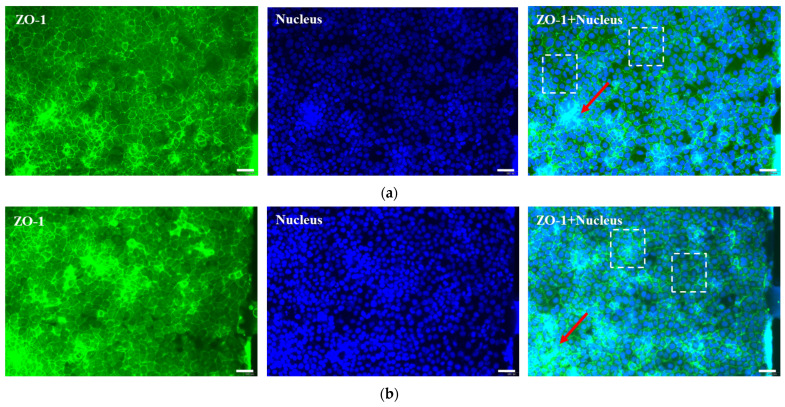
Characterization of the model with the representative immunofluorescent max projections (20× magnification) at the cultivation time of (**a**) 24 h and (**b**) 48 h, stained for ZO-1 (green), and nucleus (blue). Scale bars: 50 μm.

The physiological relevance and functionality of the simulated intestinal microenvironment were investigated by examining the expression of key protein markers in the chip-based model via immunofluorescence staining. Specifically, the formation of functional tight junctions within the intestinal barrier was confirmed by detecting ZO-1 protein expression. As shown in Figure 3a,b, immunofluorescence images revealed that the model developed well-defined 3D cellular structures (indicated by arrows) after 24 h and 48 h of culture, with clear ZO-1 expression. After 24 h of culture, the intestinal barrier began to form within the Caco-2 channels; after 48 h, the intestinal barrier had developed into a highly intact structure. Furthermore, intestinal epithelial cells within the barrier displayed a compact and uniform morphology (dashed boxes).

### 2.3. Reconstituting Intestinal Inflammation and Injury on Chip

**Figure 4:**
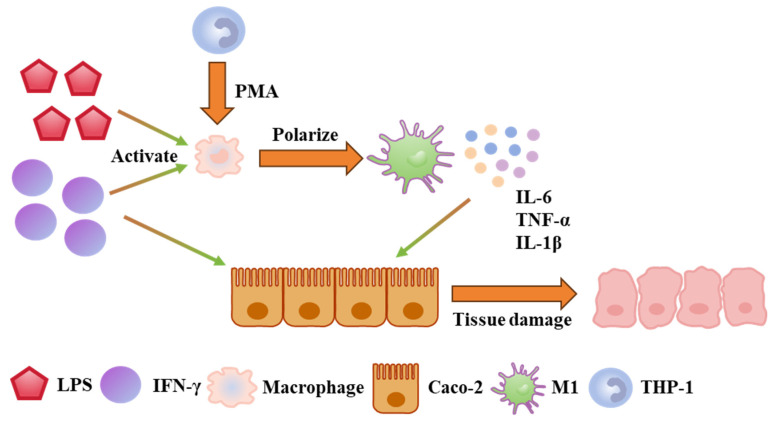
Schematic diagram illustrating the mechanisms of action of various cells in IBD.

The putative cellular mechanisms underlying IBD pathogenesis are summarized in Figure 4. Upon stimulation with lipopolysaccharides (LPSs) and interferon-gamma (IFN-γ), M0 macrophages undergo activation and polarize into the pro-inflammatory M1 phenotype [35]. These M1 macrophages subsequently secrete high levels of pro-inflammatory cytokines, including IL-6, TNF-α, and IL-1β. These cytokines compromise the intestinal barrier integrity, leading to disruption of tight junctions and altered expression of ZO-1, which collectively exacerbate IBD pathology [36,37,38].

In total, 300 μL of a pro-inflammatory cocktail of 500 ng/mL LPS and 100 ng/mL IFN-γ was added to the inlets of the two lateral channels of the intestinal-on-a-chip. At 24 h and 48 h post-stimulation, the Caco-2 cell status and intestinal barrier function were assessed via live/dead staining, immunofluorescence, and permeability measurement using a 70 kDa fluorescein isothiocyanate (FITC)–dextran probe.

**Figure 5:**
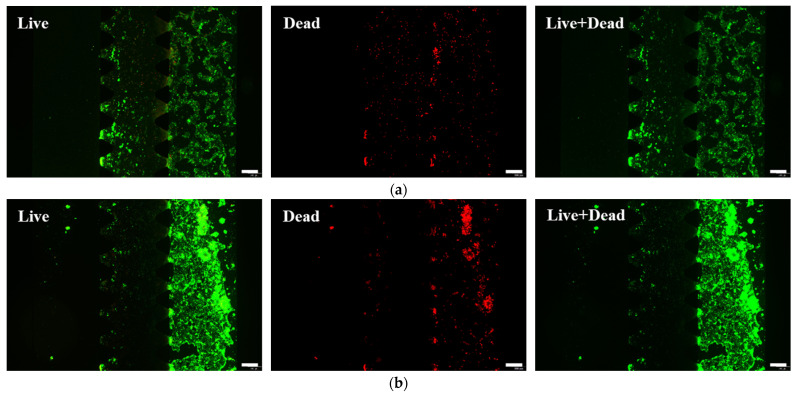

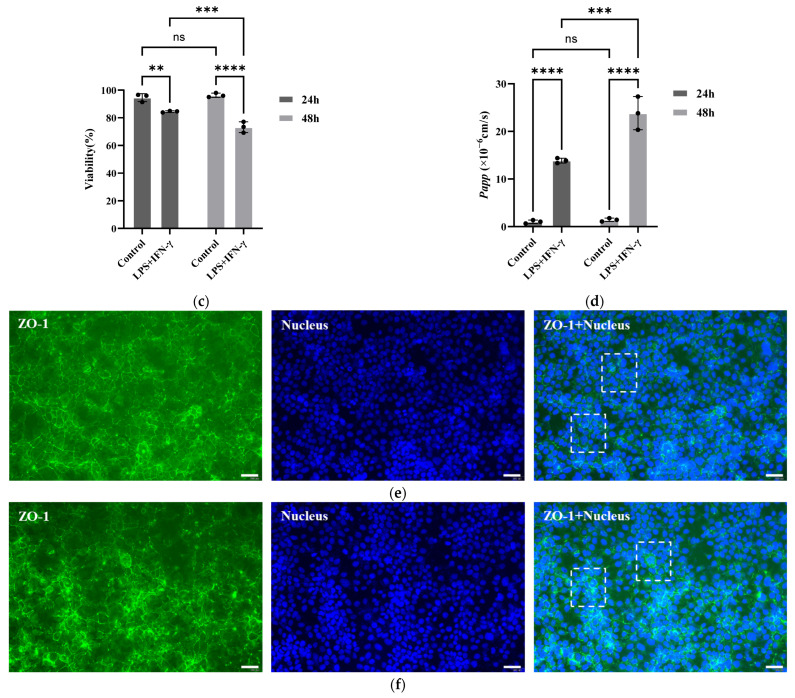
Representative images of live and dead cell staining after exposure to LPS and IFN-γ stimulation for (**a**) 24 h and (**b**) 48 h. Live cells with calcein-AM and dead cells with propidium iodide. Scale bars: 200 μm. (**c**) Cell viability of Caco-2 for 24 h and 48 h stimulation by inflammation. Data were acquired from the intestinal epithelial cell channel of three individual models (n = 3). (**d**) The model permeability for 70 kDa FITC–dextran after exposure to LPS and IFN-γ stimulation for 24 h and 48 h, shown as *Papp* values (n = 3, the intestinal epithelial cell channels of 3 individual models). Values shown in (**c**,**d**) are means ± SD and statistically compared by two-way ANOVA, respectively (ns: not significant; **: *p* < 0.01; ***: *p* < 0.001; and ****: *p* < 0.0001). Characterization of the model with the representative immunofluorescent max projections (20× magnification) after exposure to LPS and IFN-γ stimulation for (**e**) 24 h and (**f**) 48 h. Scale bars: 50 μm.

As shown in Figure 5a,b, live/dead staining revealed a marked reduction in Caco-2 cell viability following inflammatory stimulation, which became substantially more pronounced at 48 h compared to 24 h. Quantitative analyses confirmed this time-dependent decrease in viability, as shown in Figure 5c. Cell viability declined to 85.36% at 24 h post-stimulation and further decreased to 74.39% at 48 h. This progressive cell death suggests continuous M0 macrophage activation and consequent aggravation of the inflammatory response.

As shown in Figure 5d, permeability assessment using the FITC–dextran probe showed a time-dependent increase in barrier leakage. The apparent permeability (*Papp*) coefficient rose to 13.86 × 10^−6^ cm/s at 24 h and further increased to 23.82 × 10^−6^ cm/s at 48 h post-stimulation. These values were significantly higher than those in the control group, indicating severe barrier disruption. The elevated permeability at 24 h confirmed significant impairment, allowing paracellular dextran flux. By 48 h, barrier disruption was exacerbated, demonstrating a progressive deterioration of barrier function under sustained M1 macrophage activity.

As shown in Figure 5e,f, immunofluorescence analysis of the inflammation-induced model corroborated that the intestinal barrier was significantly compromised. The intestinal barrier displayed severe structural compromise, characterized by absent ZO-1 expression and underdeveloped 3D architecture. Notably, the presence of giant multinucleated cells was observed at 48 h (dashed boxes).

### 2.4. Drug Evaluation on Chip

To validate the utility of this model for drug evaluation, infliximab, a first-line therapeutic for IBD, was selected for testing. Infliximab is a monoclonal antibody that specifically neutralizes TNF-α [39,40,41]. It was the first anti-TNF-α drug approved by the U.S. Food and Drug Administration (FDA) for moderate-to-severe IBD and remains a cornerstone therapy. It exerts its therapeutic effect by binding with high affinity to both soluble and transmembrane TNF-α, leading to neutralization. This interaction blocks downstream pro-inflammatory signaling, inhibits immune cell activation and inflammatory cytokine release, and reduces intestinal epithelial cell apoptosis. Collectively, these actions attenuate tissue inflammation and injury, thereby promoting the restoration of intestinal homeostasis [42,43,44].

Following 48 h of inflammatory priming with LPS and IFN-γ, the established IBD model was treated with 300 μL of 10 μg/mL infliximab, which was added to the inlets of two lateral channels for 48 h under continuous perfusion. To assess cellular status within the chip and intestinal barrier function, the models were subjected to live/dead staining, a 70 kDa FITC–dextran permeability assay, immunofluorescence, and secretion analysis of three inflammatory factors (IL-6, TNF-α, and IL-1β) after 24 h and 48 h of continuous drug exposure.

**Figure 6:**
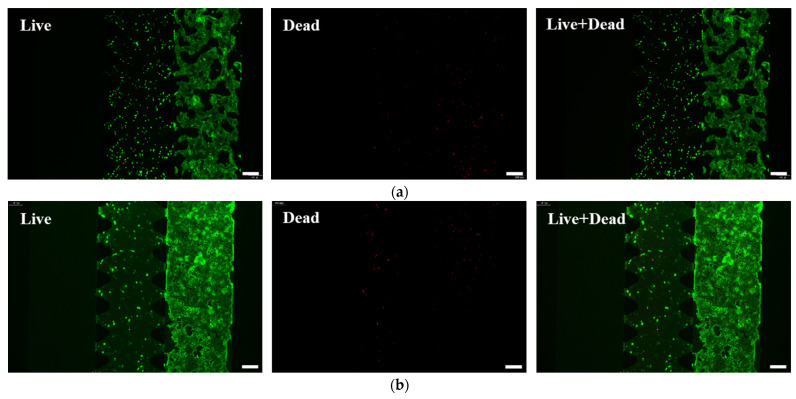

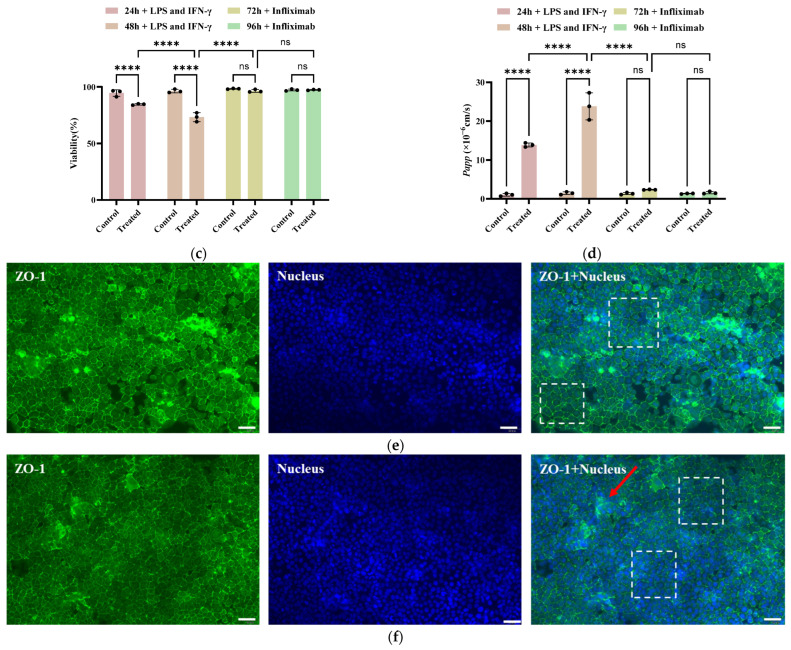
Representative images of live and dead cell staining after treatment with infliximab for (**a**) 24 h and (**b**) 48 h. Live cells with calcein-AM and dead cells with propidium iodide. Scale bars: 200 μm. (**c**) Cell viability of Caco-2. (**d**) The model’s permeability for 70 kDa FITC–dextran, which is shown as *Papp* values, was assessed in the triggered (treated) and non-triggered (control) conditions 48 h after exposure to the LPS and IFN-γ and then after treatment with infliximab for 48 h. Data were acquired from the intestinal epithelial cell channel of three individual models (n = 3). Values are means ± SD. Statistical analysis by two-way ANOVA (ns: not significant; ****: *p* < 0.0001). Characterization of the model with the representative immunofluorescent max projections (20× magnification) after treatment with infliximab for (**e**) 24 h and (**f**) 48 h. Scale bars: 50 μm.

As shown in Figure 6a,b, live/dead staining demonstrated that infliximab treatment markedly reduced Caco-2 cell death at 24 h, with near-complete inhibition observed by 48 h. As shown in Figure 6c, quantitative analysis corroborated these findings. At 24 h post-treatment, cell viability recovered to 96.47%, showing no significant difference from the control group (98.37%). This indicates a pronounced therapeutic effect of infliximab. The restored viability suggests that the drug may mitigate inflammation, potentially by suppressing M1 macrophage activation and/or promoting an M2 phenotype, thereby improving the local microenvironment. After 48 h of treatment, viability in the infliximab group further increased to 97.37%, even slightly surpassing the control value (97.29%). This suggests that prolonged infliximab exposure may confer additional benefits for epithelial integrity, possibly via mechanisms beyond immediate anti-inflammatory effects.

As shown in Figure 6d, the *Papp* coefficient decreased to 2.35 × 10^−6^ cm/s at 24 h and further declined to 1.56 × 10^−6^ cm/s at 48 h post-treatment. These values represented a significant reduction compared to the inflammation-only group and were comparable to those under normal culture conditions. This indicates that under the treatment of infliximab, the intestinal barrier is gradually repaired, tight junctions are restored, and intestinal permeability is consequently reduced.

As shown in Figure 6e,f, immunofluorescence analysis of ZO-1 corroborated that following treatment with infliximab, the intestinal barrier was significantly repaired, evidenced by the re-establishment of normal ZO-1 expression and the reappearance of a typical three-dimensional cellular structure by 48 h (arrow). Furthermore, the intestinal epithelial cells exhibited a compact, uniform morphology and were neatly arranged (dashed boxes).

**Figure 7:**
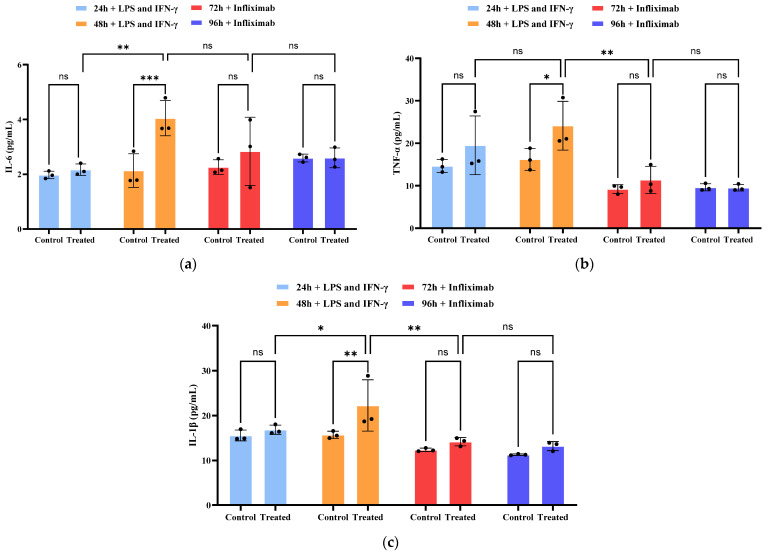
Secretion of the proinflammatory cytokine. (**a**) IL-6, (**b**) TNF-α, and (**c**) IL-1β were assessed in the triggered (treated) and non-triggered (control) conditions 48 h after exposure to LPS and IFN-γ and then after treatment with infliximab for 48 h. Data were acquired from the intestinal epithelial cell channel of three individual models (n = 3). Values are means ± SD. Statistical analysis by two-way ANOVA (ns: not significant; *: *p* < 0.05; **: *p* < 0.01; ***: *p* < 0.001).

As shown in Figure 7a–c, IL-6, IL-1β, and TNF-α levels were measured in the supernatant samples collected from the chips after inflammatory stimulation and drug treatment. In the inflammation-only group, cytokine levels exhibited a modest increase at 24 h post-stimulation. By 48 h, a marked and significant upregulation of all three cytokines was observed, confirming the establishment of a robust inflammatory milieu. Infliximab treatment potently suppressed this cytokine surge. After 24 h of drug exposure, cytokine levels were significantly reduced. Following 48 h of treatment, they were effectively restored to baseline levels, showing no statistically significant difference from the control group. Collectively, these data demonstrate that infliximab effectively inhibits the secretion of pro-inflammatory cytokines in this intestinal-on-a-chip IBD model, effectively attenuating the inflammatory cascade.

## 3. Discussion

This study established a biomimetic intestinal-on-a-chip model that recapitulates key aspects of the intestinal inflammatory microenvironment, providing a novel platform for IBD research. The model employs established cell lines (Caco-2- and THP-1-derived macrophages) co-cultured within a three-channel microarchitecture on a collagen type I extracellular matrix (ECM). This design incorporates immunocompetent M0 macrophages within a central stromal compartment, enabling direct macrophage–epithelial crosstalk that mirrors the in vivo immune–epithelial interface. Robust cellular adhesion within the microchannels facilitated the formation of a stable, physiologically relevant intestinal barrier with functional tight junctions.

The system generates physiologically relevant fluid shear stress via a passive gravity-driven flow mechanism, obviating the need for external pumps and simplifying operational complexity. Application of the pro-inflammatory agonists LPS and IFN-γ to the macrophage compartment successfully induced a disease-relevant phenotype, characterized by the release of pro-inflammatory cytokines (IL-6, IL-1β, and TNF-α) and a degradation of intestinal barrier integrity. Temporal analysis revealed that inflammatory stimulation triggered sustained macrophage activation, resulting in a time-dependent exacerbation of barrier dysfunction and cytokine storm—a hallmark of progressive IBD. Thus, the platform provides a controllable and efficient in vitro system for modeling intestinal inflammation.

Furthermore, in contrast to prior studies on intestinal-on-a-chip, this model enables the introduction of more immune cells to study their role in IBD. Importantly, to demonstrate the platform’s utility in translational research, we evaluated its response to the therapeutic antibody Infliximab, thereby underscoring its potential as a robust tool for preclinical drug testing and development. It offers high reproducibility and operational simplicity, thereby opening new avenues for mechanistic inquiry that complement and, in some aspects, surpass the capabilities of traditional animal models. Consequently, the integration of such microfluidic models into pre-clinical drug development pipelines could enhance compound screening efficiency, improve patient-specific therapeutic predictions, and potentially reduce the high attrition rates and costs associated with conventional drug discovery.

However, in its current state, the absence of primary cells or patient-derived intestinal organoids further limits the physiological relevance and translational potential of the model. As a result, while the system is suitable for demonstrating proof-of-concept microfluidic interactions and inflammatory responses, its ability to faithfully mimic in vivo intestinal immune–epithelial crosstalk is still limited. In addition, our study can not comprehensively evaluate intestinal barrier integrity because of the absence of complementary functional readouts. Measuring transepithelial electrical resistance (TEER) would be useful for a more comprehensive assessment of the intestinal barrier.

## 4. Materials and Methods

### 4.1. Cell Culture

Caco-2 Cell: The human colon adenocarcinoma cell line Caco-2 was cultured in a humidified incubator at 37 °C with 5% CO_2_ and 90% relative humidity. Dulbecco’s modified Eagle’s medium (DMEM) (Sangon Biotech, Shanghai, China) containing 4.5 g/L glucose medium (DMEM/HG) supplemented with 10% (*w*/*v*) fetal bovine serum (FBS) and 1% penicillin-streptomycin (PS) was used as the growth medium. The culture medium was refreshed every two days.

THP-1 Cell: The human acute monocytic leukemia cell line THP-1 was cultured in a Petri dish in Roswell Park Memorial Institute 1640 medium (Sangon Biotech, Shanghai, China) supplemented with 10% FBS and 1% PS. THP-1 cells cultured in a Petri dish were differentiated toward macrophages by exposure to 100 ng/mL PMA (phorbol 12-myristate 13-acetate) in the medium. After 24 h, the medium was replaced with a standard culture medium, and the cells were further incubated for 24 h and then trypsinized with TrypLE Express for seeding in the chip.

### 4.2. Fabrication of the Microfluidic Device

The intestinal-on-a-chip was designed with AUTOCAD 2020 software (AUTODESK, San Francisco, CA, USA), and the design was printed as an acetate mask. Master molds were obtained in silicon wafers using standard photolithography techniques in a clean room environment. The master molds were fabricated using a two-step spin-coating process. Two layers of negative photoresist SU-8 were sequentially applied, achieving a total height of 130 μm. Following spin-coating, the photoresist was first soft baked (65 °C for 5 min and then 95 °C for 10 min) and then exposed through an acetate mask using an I-line mask aligner to transfer the microchannel pattern. Subsequently, the wafers underwent post-exposure baking, were developed for 10 min, and then hard-baked at 65 °C for 10 min and then at 95 °C for 30 min. Finally, the wafers were silanized for 1 h with trifluorosilane. The PDMS replicas were obtained using the well-established method of soft lithography [45,46]. PDMS monomer and curing agent were mixed in a ratio of 10:1 and poured into Petri dishes containing the mold and cured for 2 h at 80 °C. To achieve irreversible bonding, each chip was plasma-treated in high mode for 30 s and then bonded to a glass slide. Finally, the assembled chip was cured overnight in an oven at 70 °C. This chip is disposable.

### 4.3. Immunohistochemical Analysis

Cells were cultured on the chip and washed three times with phosphate-buffered saline (PBS), with a 3 min interval between each wash, and then fixed with 4% paraformaldehyde in phosphate-buffered saline for 10 min, followed by three PBS washes at 3 min intervals. Permeabilize with PBS containing 0.1% Triton X-100 (Sangon Biotech, Shanghai, China) for 10 min and then wash three times with PBS at 3 min intervals. Block with PBS containing 1% bovine serum albumin at room temperature in the dark for 1 h. Incubate with ZO-1 rabbit polyclonal antibodies (Thermo Fisher Scientific, Waltham, MA, USA) at a concentration of 1 μg/mL for 3 h at room temperature; wash three times with PBS at 3 min intervals and then incubate with a Goat anti-Rabbit IgG (Heavy Chain) Super clonal Secondary Antibody (Thermo Fisher Scientific, Waltham, MA, USA) at a concentration of 2 μg/mL for 1 h at room temperature. Wash three times with PBS at 3 min intervals. Finally, add DAPI staining solution (Sangon Biotech, Shanghai, China) and incubate in the dark for 5 min to stain cell nuclei. Store cells in PBS and image cells on the chip using the ImageXpress Micro XLS—High Content Imaging System.

### 4.4. Induction of Inflammation

To induce intestinal inflammation, the model was administered 500 ng/mL of lipopolysaccharides (LPSs) derived from *Escherichia coli* serotype O111:B4 (Merck, Darmstadt, Germany) apically to mimic Gram-negative bacterial endotoxin exposure in the intestinal lumen. Simultaneously, human recombinant interferon-γ (IFN-γ; Peprotech, Rocky Hill, NJ, USA) was introduced into the same channel of the model at a final concentration of 100 ng/mL.

### 4.5. Cytokine Secretion Analysis

For the analysis of inflammatory cytokines, culture medium from both apical and basolateral channels was collected, and samples from the corresponding inlet and outlet ports were pooled. The pooled samples were immediately snap-frozen in liquid nitrogen and stored at −80 °C until subsequent analysis. Quantification of IL-6, TNF-α, and IL-1β levels in each sample (n = 3) was performed using enzyme-linked immunosorbent assay (ELISA) kits (Thermo Fisher Scientific, Waltham, MA, USA) in strict accordance with the manufacturer’s protocols.

### 4.6. Cell Viability Staining

Using the calcein-AM/propidium iodide live/dead staining kit (Thermo Fisher Scientific, Waltham, MA, USA), the chip was washed three times with PBS before staining with the calcein-AM/propidium solution. It was then incubated at 37 °C in the dark for 20 min. Viable cells exhibited green fluorescence, while dead cells stained red.

### 4.7. Fluorescence Imaging Permeability Assay

Barrier permeability was assessed using fluorescence tracers with dextran70-FITC (70 kDa, D70). In previous studies, these standard tracers have been widely used to validate the permeability of the intestinal barrier, with validation based on the size-exclusion effect [47,48,49]. A 100 µM/mL fluorescent tracer solution was prepared in the DMEM medium to maintain physiological conditions. Prior to the assay, the Caco-2 channel was rinsed with PBS to remove debris, and both channels were supplied with DMEM medium. A baseline image (time point 0) was captured using an inverted fluorescence microscope (Nikon Eclipse Ti2, Nikon Precision, Shanghai, China) under both bright-field and fluorescent settings. The tracer solution was then injected into the Caco-2 channel, while the other lateral channel only contained DMEM medium. Time-lapse fluorescence images were acquired at 3 min intervals for 30 min. The apparent permeability coefficient (*Papp*, cm/s) was calculated from the acquired images using Fiji/ImageJ^®^ 1.54p software, and the *Papp* is determined as detailed by Campisi and collaborators [50,51,52]:

(1) $$Papp=\left(\frac{1}{\left(Et1-Ct1\right)}\right)\times \left(\frac{\left(Ct2-Ct1\right)}{t}\right)\times \frac{V_{c}}{S}$$

where *Et*1 and *Ct*1 are the fluorescence intensity at the initial time in the Caco-2 channel and central channel, respectively (u.a.); *Ct2* is intensity in the central channel at end time (u.a.); *t* is the difference between the initial and the final time (s); *V_c_* is the volume of the central channel (cm^3^); S is the surface area of the epithelial wall (cm^2^).

### 4.8. Statistics and Data Analysis

All data were analysed by averaging the values of at least three microfluidic devices, with each device representing one independent experiment. All results and error bars in this article were represented as the mean ± SD. All statistics were conducted with the Graphpad prism 10^®^ software (GraphPad Software Inc., San Diego, CA, USA). Finally, all data were organized for visualization by Adobe Illustrator (Adobe Inc., San Jose, CA, USA) using input files from the aforementioned software.

## 5. Conclusions

The intestinal-on-a-chip model developed herein systematically recapitulates the progression of intestinal inflammation, rendering it suitable for a spectrum of applications from mechanistic investigation to screening of anti-inflammatory compounds in preclinical development. Its controlled microenvironment, a feature unattainable in traditional models, enables the precise dissection of immune cell-mediated pathogenesis in IBD. This positions the platform as an efficient tool for drug screening and therapeutic strategy development. In summary, this platform—with its flexibly configurable, biomimetic architecture and rigorously validated disease phenotype—represents a significant advance over conventional in vitro models. It constitutes a promising and translatable tool for elucidating IBD mechanisms and accelerating the development of personalized therapeutics.

## Data Availability

The original contributions presented in this study are included in the article. Further inquiries can be directed to the corresponding authors.
